# MicroRNA-126a-5p enhances myocardial ischemia-reperfusion injury through suppressing Hspb8 expression

**DOI:** 10.18632/oncotarget.21613

**Published:** 2017-10-07

**Authors:** Bimei Jiang, Yanjuan Liu, Pengfei Liang, Yuanbin Li, Zhenguo Liu, Zhongyi Tong, Qinglan Lv, Meidong Liu, Xianzhong Xiao

**Affiliations:** ^1^ Department of Pathophysiology, Xiangya School of Medicine, Central South University, Changsha, P. R. China; ^2^ Department of Burns and Plastic Surgery, Xiangya Hospital, Central South University, Changsha, P. R. China; ^3^ Dorothy M. Davis Heart and Lung Research Institute, Division of Cardiovascular Medicine, Department of Internal Medicine, The Ohio State University Wexner Medical Center, Columbus, OH, USA

**Keywords:** microRNA-126a-5p, myocardium, ischemia-reperfusion injury, Hspb8, H9C2 cells

## Abstract

Previously, we found several genes are involved in myocardial ischemia-reperfusion (M-I/R) injury. In this report, we first developed a mouse model of M-I/R injury and demonstrated microRNA-126a-5p was associated with the M-I/R injury by using high-throughput microRNA expression analysis. We further investigated the expression and function of microRNA-126a-5p during mouse M-I/R injury. We observed high expression of microRNA-126a-5p in the M-I/R mice and increased levels of LDH and CK-MB (damage markers) in the serum. H_2_O_2_ and hypoxia/reoxygenation (H/R) treatment significantly increased the expression of microRNA-126a-5p in H9C2 cells in concentration- and time-dependent manners. Moreover, microRNA-126a-5p overexpression in H9C2 cells inhibited cell viability but increased LDH release and caspase 3 activity. Cardiac function analysis based on the measurements of hemodynamic parameters showed that microRNA-126a-5p expression ablation in M-I/R injured mice led to the reversal of the symptoms caused by M-I/R injury. Transesophageal echocardiography also revealed that the values of LVIDd and LVIDs were decreased while the values of LVFS% and LVEF% were increased in M-I/R injured mice after treatment with microRNA-126a-5p inhibitor, compared with the M-I/R injured mice treated with the control. Bioinformatic analysis demonstrated that Hspb8, a protective protein in myocardium, was the target of microRNA-126a-5p. Thus, these findings indicated that microRNA-126a-5p was up-regulated in mouse M-I/R model and promoted M-I/R injury *in vivo* through suppressing the expression of Hspb8, which may shed light on the development of potential therapeutic target for M-I/R injury.

## INTRODUCTION

Ischemic heart disease (IHD) contributes to the development of cardiovascular disease, which in turn leads to patient death. The current IHD treatment involves reperfusion of the blocked artery, but reperfusion may lead to myocardial ischemia-reperfusion (M-I/R) injury, including myocardial stunning, cardiomyocyte death, microvascular obstruction and arrhythmias [1]. Therefore, it is important to figure out potential protective mechanisms for myocardial I/R-induced injury.

MicroRNAs are a kind of endogenous, single-stranded RNAs about 18-20 nucleotides, which can regulate gene expression by targeting 3′-end untranslated region (UTR) of mRNAs at posttranscriptional level. It has been speculated that more than 60% of human genes are regulated by microRNAs [2–4]. Number of studies have reported the involvement of microRNAs in the regulation of multiple important physiological and pathological processes [5–7], including cell proliferation, migration and apoptosis. Some microRNAs such as microRNA-320, microRNA-103/107, microRNA-141 and microRNA-21 have specifically been implicated to play an important role in the cardiac development and myocardial I/R injury [8–12].

Among various microRNAs, microRNA-126a-5p is abundantly expressed in the endothelial cells and tissues with lots of vessels, such as lung, liver, and heart [13]. Its association with coronary heart disease, heart failure and atherosclerosis has been reported [14,15]. However, there is no study about the relationship between microRNA-126a-5p expression and M-I/R injury. Therefore, in this study, we performed a comparative microRNA profiling to identify the differentially expressed miRNAs in mouse M-I/R injury model and identified microRNA-126a-5p as a promising candidate. Further investigation showed that microRNA-126a-5p was up-regulated in mouse M-I/R model and promoted M-I/R injury *in vivo* through suppressing the expression of Hspb8, a protective protein in cardiomyocytes. These findings provide strong insights for developing a new therapeutic strategy for M-I/R injury.

## RESULTS

### Analysis of microRNA-126a-5p expression during M-I/R injury

**Figure 1:**
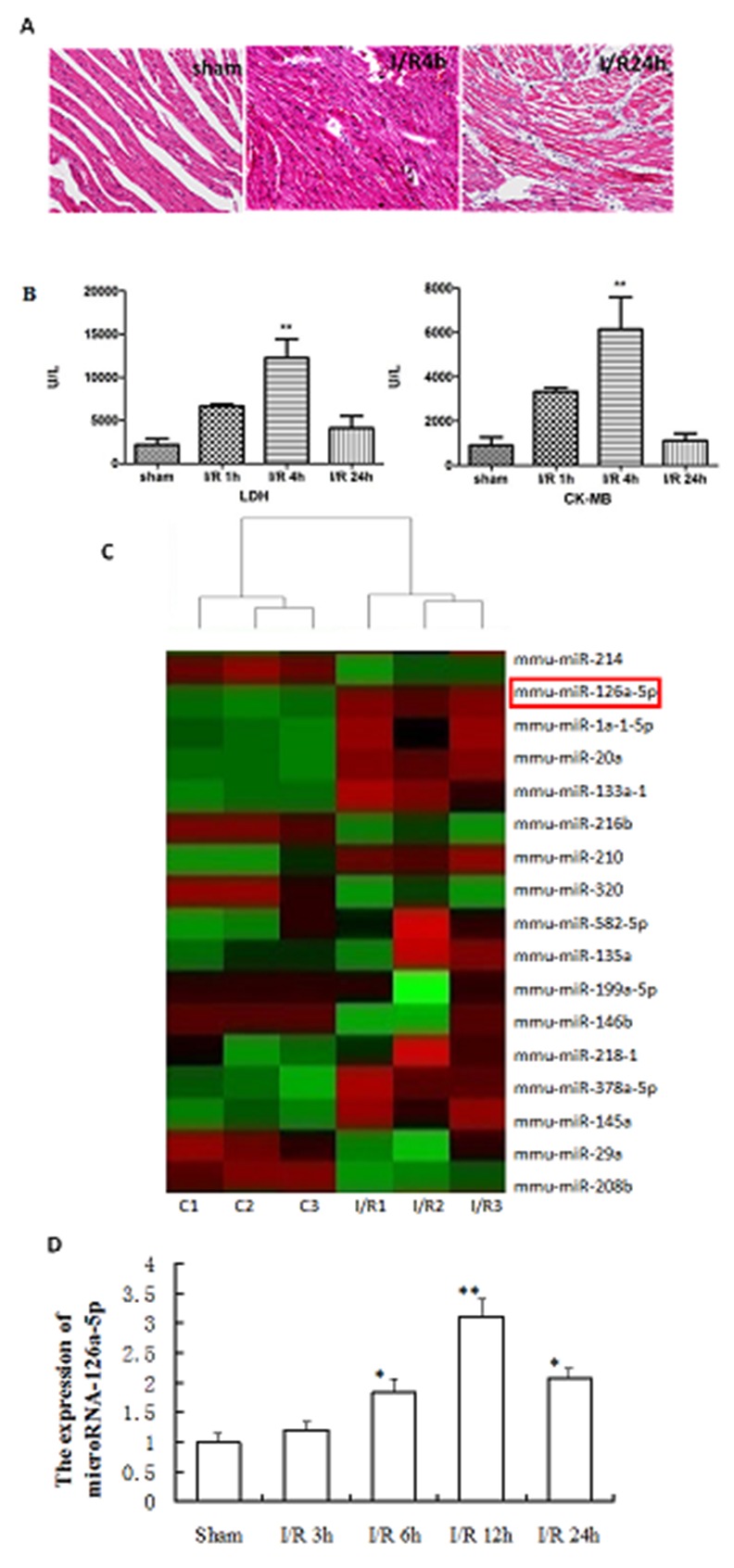
Analysis of microRNA-126a-5p expression during myocardial ischemia reperfusion injury **(A)** HE staining displaying the change in myocardial tissue morphology. sham: sham group, I/R: ischemia reperfusion injury. **(B)** Detection of LDH and CK-MB levels in serum to assess myocardial damage. LDH: lactate dehydrogenase; CK-MB, creatine kinase-MB. ^**^, *P* < 0.01, *vs*. sham group, n=6. **(C)** Heatmap of microarray result. (Simplified version). Red color represents up-regulation while green color represents down-regulation. **(D)** RT-QPCR analysis of the microRNA-126a-5p expression in the myocardium from sham and ischemia reperfusion injury mice. ^*^, *P* < 0.05, *vs*. sham group; ^**^, *P* < 0.01, *vs*. sham group, n=6.

To study the expression profile of microRNAs during M-I/R injury, a M-I/R injury mouse model was established. As shown in Figure 1, we found myocardial fibers were disorderly arranged and dissolved, along with being infiltrated by the inflammatory cells (Figure 1A). Moreover, the mice from M-I/R group displayed higher serum levels of LDH and CK-MB than the sham group, especially 4 hrs after reperfusion (*P* < 0.01) (Figure 1B). These results suggest the M-I/R injury mouse model was established successfully. Then, a miRNA microarray assay was performed in the myocardial tissues harvested at 12 h after reperfusion. According to the dramatical changes in the heat-map results, microRNA-126a-5p was one of the top upregulated microRNAs in all samples (Figure 1C). To verify the results of microarray, qPCR results further confirmed that microRNA-126a-5p was significantly upregulated at the 6 h after reperfusion and reached to the peak at 12 hrs after reperfusion (Figure 1D). These data suggest that the elevated expression of microRNA-126a-5p may play important roles in M-I/R injury.

### The role of microNA-126a-5p in H9C2 cells injury induced by H_2_O_2_ or H/R

**Figure 2:**
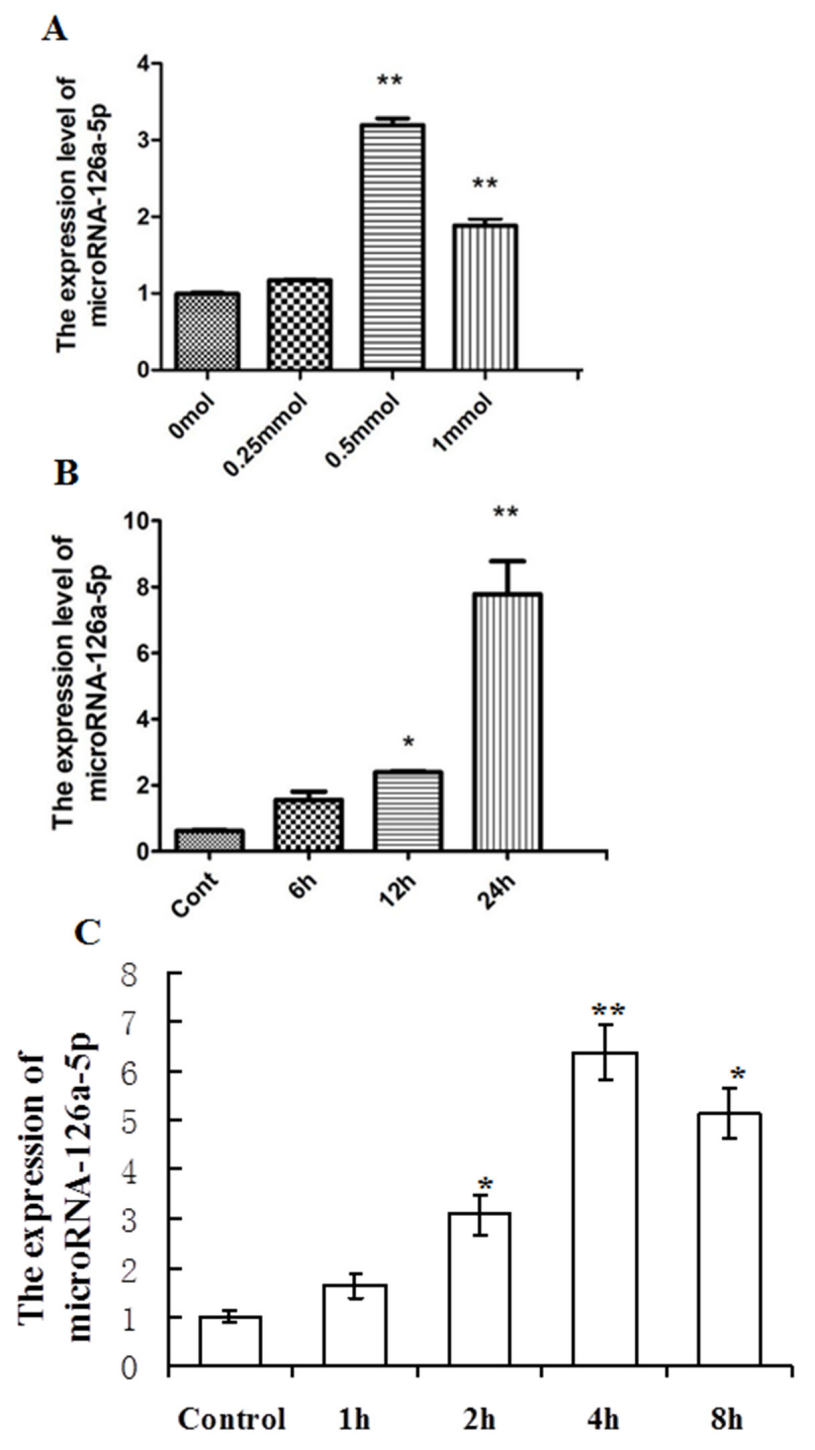
Analysis of microRNA-126a-5p expression in H9C2 cardiomyocytes injury mediated by H_2_O_2_ or hypoxia/reoxygenation (H/R) **(A)** H9C2 cells treated with H_2_O_2_ at different concentrations, 0 mmol/L, 0.25 mmol/L, 0.5 mmol/L, 1 mmol/L for 24 hrs, microRNA-126a-5p expression was analyzed by RT-QPCR. ^**^, *P* < 0.01, *vs*. 0 mmol group, n=6. **(B)** H9C2 cells treated with 0.5 mmol/L H_2_O_2_ for 0h, 6h, 12h, 24h, and microRNA-126a-5p expression was assessed by RT-QPCR. ^*^, *P* < 0.05 *vs*. control group, n=8, ^**^, *P* < 0.01 *vs*. 0h group, n=8. **(C)** After H9C2 cells were exposed to 1h,2h,4 h, 8h of Na_2_S_2_O_4_ and then to 12 h of reoxygenation, microRNA-126a-5p expression was analyzed by RT-QPCR. ^*^, P<0.05 vs. control group; ^**^, P<0.01 vs. control group, n=6.

To further examine the *in vitro* expression of microRNA-126a-5p, H9C2 cells were treated with various concentrations of H_2_O_2_ for different time points to induce cell damage, then the expression of microRNA-126a-5p was assessed by RT-QPCR. As shown in Figure 2, H_2_O_2_ treatment increased the expression of microRNA-126a-5p in H9C2 cells and 0.5 mmol/L of H_2_O_2_ treatment for 24 hrs showed the maximum effect. Moreover, the increase in microRNA-126a-5p expression by H_2_O_2_ treatment was time-dependent (Figure 2A, 2B). Further, the H9C2 cells were treated with Na_2_S_2_O_4_ (4mM) to induce hypoxia/reoxygenation (H/R) injury. As shown in Figure 2C, compared to the control group, H/R injury (hypoxia for 1, 2, 4, 8 h and reoxygenation 12 h) increased the expression of microRNA-126a-5p, and hypoxia 4 h and reoxygenation 12 h treatment showed the maximum effect.

**Figure 3:**
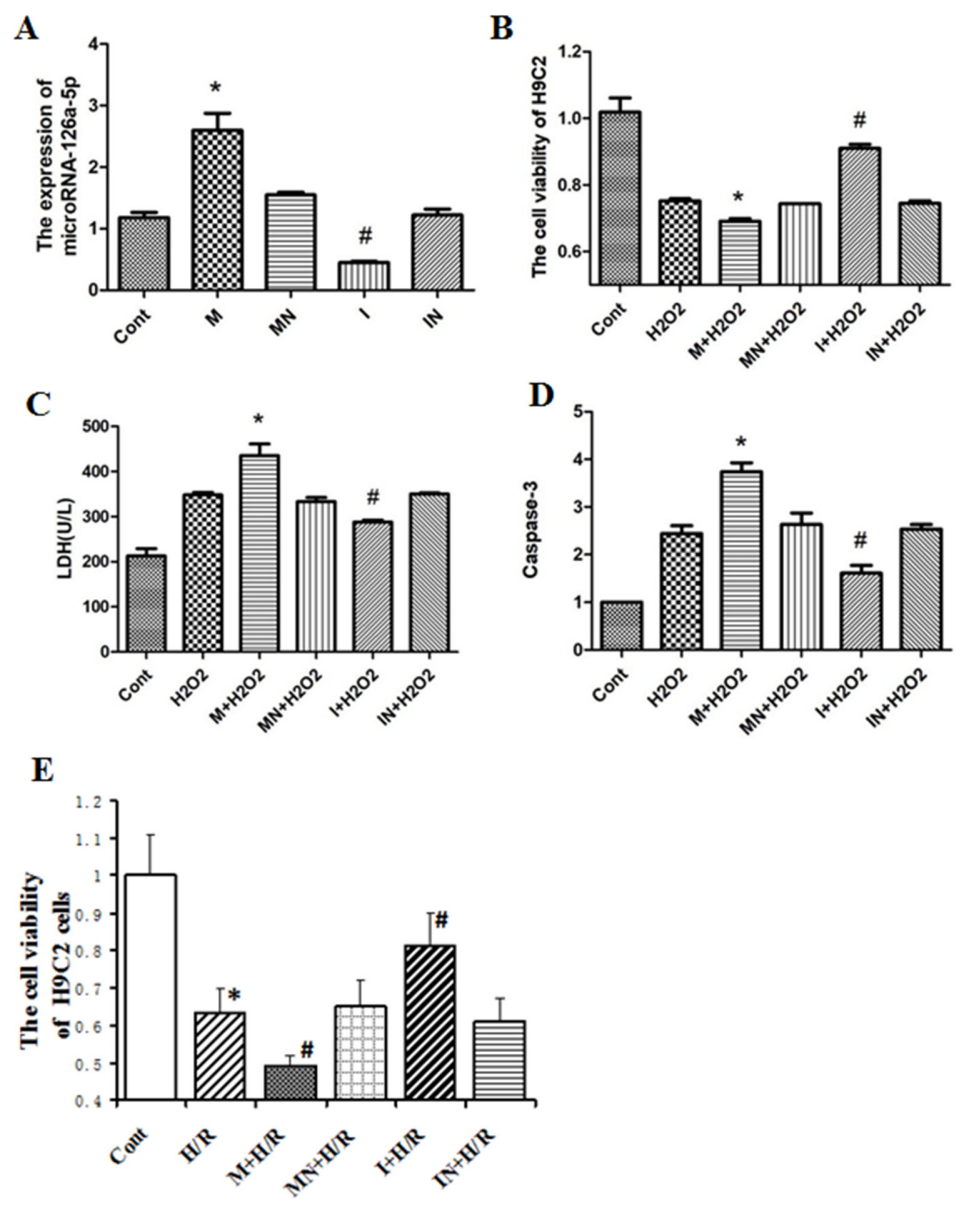
The role of microRNA-126a-5p in H9C2 cells injury mediated by H_2_O_2_ or H/R **(A)** RT-QPCR detected the expression of microRNA-126a-5p in H9C2 cells transfected with microRNA-126a-5p mimic or inhibitor. ^*^, *P* < 0.05, *vs*. control group, n=6; ^#^, *P* < 0.05, *vs*. control group, n=6. **(B)** Analysis ofcell viability of H9C2 cardiomyocytes treated with H_2_O_2_ after microRNA-126a-5p mimic or inhibitor transfection. ^*^, *P* < 0.05, *vs*. H_2_O_2_, n=8. **(C)** Analysis of microRNA-126a-5p mimic or inhibitor effects on LDH release in H_2_O_2_-treated H9C2 cardiomyocytes. ^*^, *P* < 0.05, *vs*. H_2_O_2_, n=8. **(D)** Analysis of microRNA-126a-5p mimic or inhibitor effect on Caspase 3 activity in H_2_O_2_-treated H9C2 cardiomyocytes. ^*^, *P* < 0.05, *vs*. H_2_O_2_, n=8. **(E)** After transfected with microRNA-126a-5p mimic or inhibitor, H9C2 cells were exposed to 4 h of Na_2_S_2_O_4_ and then to 12 h of reoxygenation, cell viability was analyzed by MTT. ^*^, P<0.05, vs. Cont group; ^#^, P<0.05, vs. H/R group, n=6. M: microRNA126a-5p mimic; MN: microRNA126a-5p mimic negative; I: microRNA126a-5p inhibitor; IN: microRNA-126a-5p inhibitor negative.

To further study the role of elevated microRNA-126a-5p in H9C2 cell injury induced by H_2_O_2_ or H/R, we used its mimic and inhibitor to regulate its expression. MicroRNA-126a-5p mimic enhanced its expression in H9C2 cells compared with the control and mimic-negative transfected cells (Figure 3A). Conversely, the cells transfected with microRNA-126a-5p inhibitor showed less expression compared to control and inhibitor negative transfection (*P* < 0.05) (Figure 3A). We further treated above transfected H9C2 cells with 0.5 mmol/L H_2_O_2_ for 24 hrs, then cell viability, LDH level and caspase 3 activities in the supernatant were analyzed. Cell viability was robustly decreased after H_2_O_2_ treatment. After being transfected with microRNA-126a-5p mimic, cell viability was further decreased. While microRNA-126a-5p inhibitor significantly rescued H_2_O_2_-induced decrease in cell viability in H9C2 cells (*P* < 0.05) (Figure 3B). LDH level and caspase 3 activity were significantly increased following H_2_O_2_ exposure and further enhanced in the cells transfected with microRNA-126a-5p mimic, but H_2_O_2_-induced increase in LDH level and caspase 3 activity was reversed by microRNA-126a-5p inhibitor (Figure 3C and 3D). Additionally, two control groups (mimic-negative group and inhibitor-negative group) did not show any significant differences in all parameters compared with H_2_O_2_ group (*P* > 0.05) (Figure 3B–3D). Also, the viability assay revealed that Na_2_S_2_O_4_-induced hypoxia/reoxygenation decreased cell viability. As shown in Figure 3E, the viability of the cells exposed to Na_2_S_2_O_4_ at concentrations of 4 mM for 4 h and replacing the normal medium for an additional 12 h significantly decreased to 63.11±7.13% of the control value (n=6, P<0.01). Compared to the model group, pretreatment with microRNA-126a-5p mimic, cell viability was further decreased. While pretreatment with microRNA-126a-5p inhibitor significantly attenuate hypoxia/reoxygenation-induced decrease in cell viability in H9C2 cells (P<0.05). These data demonstrate that microRNA-126a-5p enhance H9C2 cells injury induced by H_2_O_2_ or H/R.

### Effect of microRNA-126a-5p ablation on M-I/R injury *in vivo*

**Figure 4:**
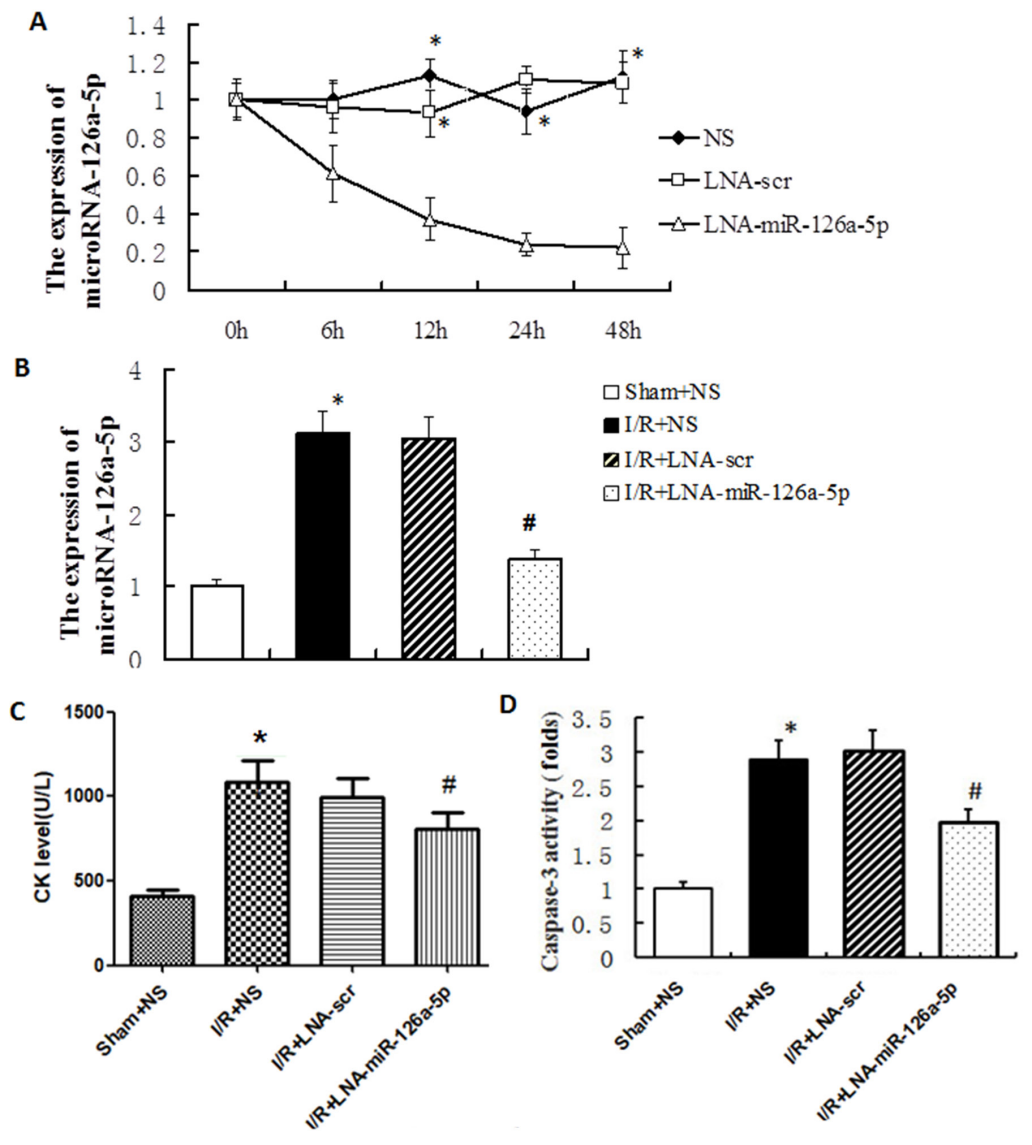
MicroRNA-126a-5p ablation alleviated the myocardial ischemia reperfusion injury *in vivo* **(A)** The microRNA-126a-5p inhibitor effectively blocked microRNA-126a-5p expression *in vivo*. ^*^, *P* < 0.01, *vs*. 0h, n=5. NS: normal saline group; LNA-microRNA-126a-5p: microRNA-126a-5p inhibitor group; LNA-scr: microRNA-126a-5p inhibitor control group. **(B)** The microRNA-126a-5p expression was inhibited by its inhibitor after 12 h of I/R injury. ^*^, *P* < 0.01, *vs*. Sham+NS group, n=8; ^#^, *P* < 0.01, *vs*. I/R+LNA-scr group, n=8. **(C)** Analysis of creatine kinase (CK) levels in mouse serum. ^*^, *P* < 0.01, *vs*. Sham+NS group, n=8; ^#^, *P* < 0.01, *vs*. I/R+LNA-scr group, n=8. **(D)** Analysis of myocardial caspase 3 activity. ^*^, *P* < 0.01, *vs*. Sham+NS group, n=8; ^#^, *P* < 0.01, *vs*. I/R+LNA-scr group, n=8. Sham+NS: sham + saline injection group; I/R+NS: ischemia reperfusion injury + saline injection group; I/R+LNA-scr: ischemia reperfusion injury + microRNA-126a-5p inhibitor control injection group; I/R+ LNA-microRNA-126a-5p: ischemia reperfusion injury + microRNA-126a-5p inhibitor injection group.

To further study the role of microRNA-126a-5p in I/R injury, we analyzed the effect of microRNA-126a-5p ablation on the M-I/R injury in mice. As shown in Figure 4, the inhibitory effect of microRNA-126a-5p inhibitor (LNA-miR-126a-5p) was obvious as early as 6 hrs after injection, in which the expression of microRNA-126a-5p was decreased by 38.53%. The expression of microRNA-126a-5p by its inhibitor was inhibited in a time-dependent manner, suggesting that microRNA-126a-5p inhibitor (LNA-miR-126a-5p) is potent and can inhibit the expression of microRNA-126a-5p effectively in mice (*P* < 0.01) (Figure 4A). We also tested this inhibitor in M-I/R injury mouse model and found that tail vein injection of microRNA-126a-5p inhibitor inhibited the up-regulation of microRNA-126a-5p expression in M-I/R injury mouse model (*P* < 0.01), while injection of control inhibitor (LNA-Scr) did not show any effect (Figure 4B). Moreover, the increased levels/expression of CK levels in serum (Figure 4C) and caspase-3 activity (Figure 4D) were inhibited by microRNA-126a-5p inhibitor in M-I/R injury mice. These results indicate that microRNA-126a-5p inhibitor can attenuate the cardiac injury.

### Effect of microRNA-126a-5p ablation on cardiac hemodynamic measurement

**Figure 5:**
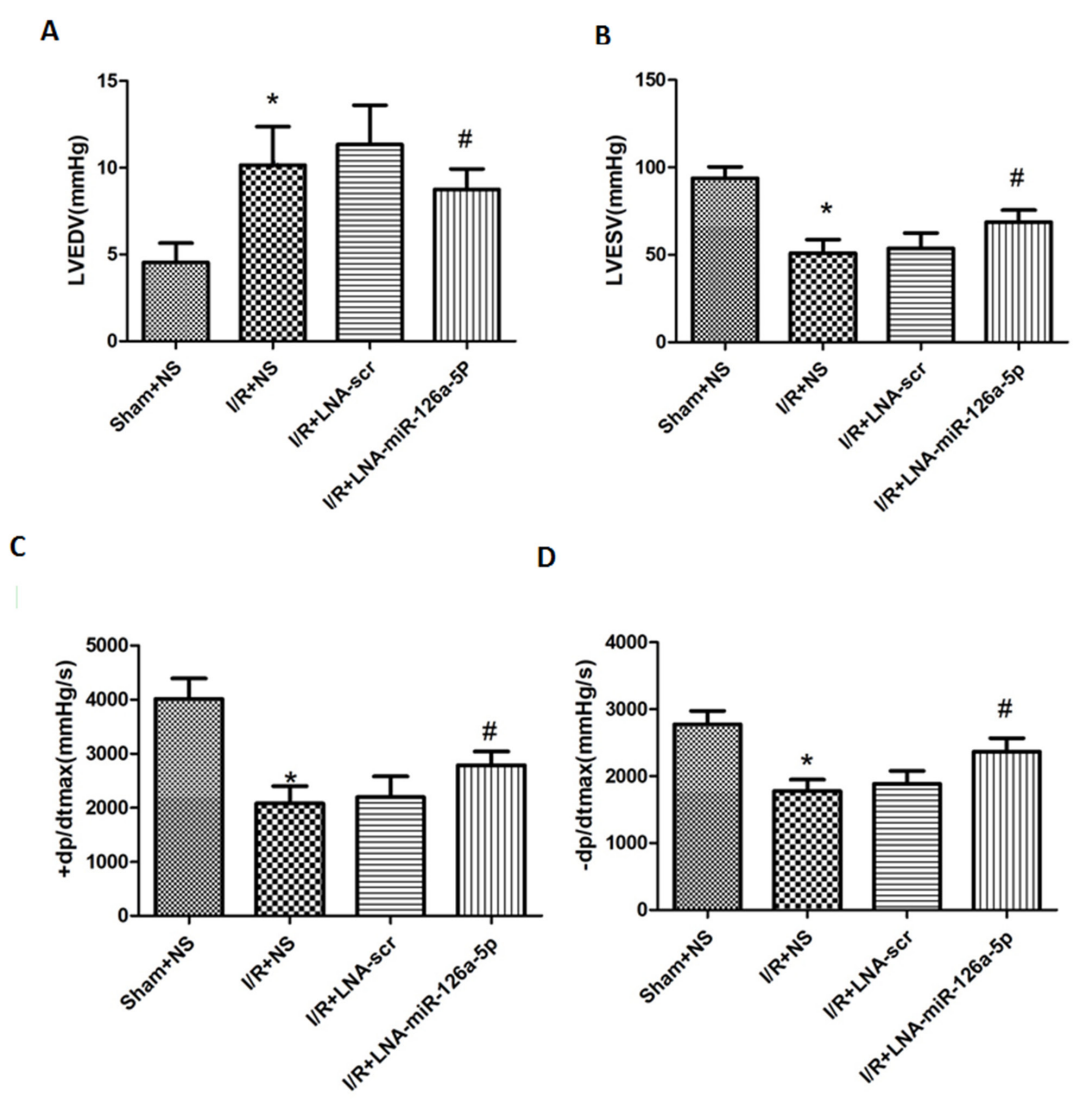
Effect of microRNA-126a-5p ablation on cardiac haemodynamic measurement Powerlab was used to detect the effect of microRNA-126a-5p inhibitor on cardiac functions in mice. LVEDP: left ventricular end-diastolic pressure. LVSP: Left ventricular systolic pressure. +dp/dt_max_: the maximum rate of left ventricular pressure rise; -dp/dt_max_, the maximum rate of left ventricle diastolic pressure change; ^*^, *P*< 0.01, *vs*. Sham+NS group, n=8; ^#^, *P* < 0.01, *vs*. I/R+LNA-scr group, n=8. Sham+NS: sham + saline injection group; I/R+NS: ischemia reperfusion injury + saline injection group; I/R+LNA-scr: ischemia reperfusion injury + microRNA-126a-5p inhibitor control injection group; I/R+ LNA-microRNA-126a-5p: ischemia reperfusion injury + microRNA-126a-5p inhibitor injection group.

The cardiac functions of the mice underwent I/R injury were assessed by measuring hemodynamic parameters. I/R injury markedly increased the LVEDP (left ventricular end-diastolic pressure) but significantly decreased the LVSP (left ventricular systolic pressure) compared to the mice in the sham group. Moreover, the +dp/dt_max_ (the maximum rate of left ventricular pressure rise) was significantly higher in I/R injury mice, but -dp/dt_max_ (the maximum rate of left ventricular pressure decline) was lower than that in the sham group. Interestingly, inhibition of microRNA-126a-5p by its inhibitor showed significant reversal of these parameters (i.e. higher LVSP and markedly lower LVEDP compared with I/R group). The +dp/dt_max_ and -dp/dt_max_ values were also reversed by the microRNA-126a-5p inhibitor (Figure 5). Additionally, the injection of scramble inhibitor showed no significant effect on I/R-induced effects on the hemodynamic parameters. Also, no significant difference in the heart rate was observed in the mice among three groups. These results indicate that inhibition of cardiac microRNA-126a-5p could improve the ventricular contractility reflected by the increase in +dp/dt_max_ and LVSP.

### Effect of microRNA-126a-5p ablation on cardiac function assessed by M-mode echocardiography

**Figure 6:**
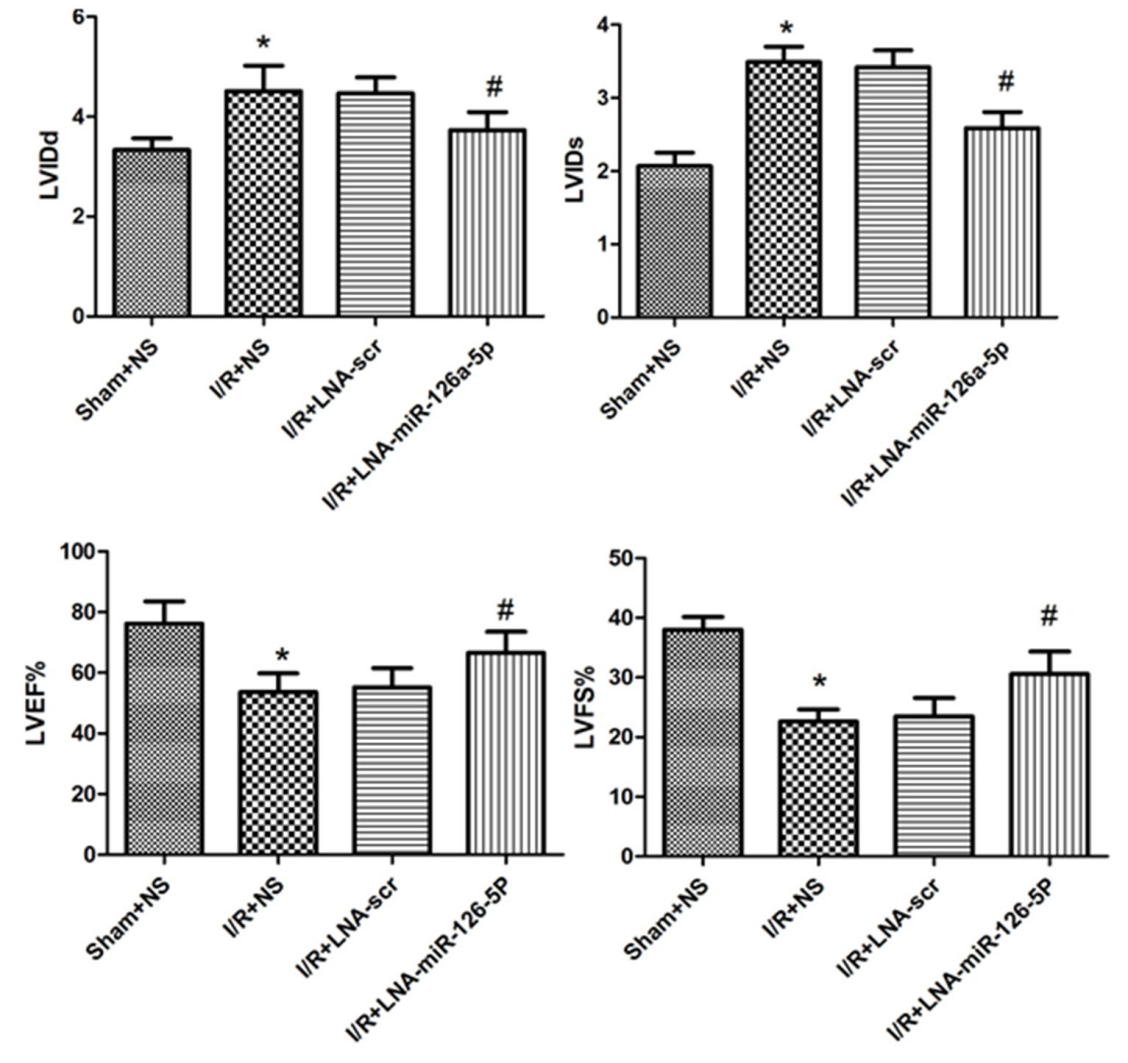
Effect of microRNA-126a-5p ablation on cardiac function by echocardiographic assessment Echocardiography was performed as described in Methods section. LVIDd: left-ventricular internal diastolic diameter; LVIDs: left-ventricular internal systolic diameter; LVEF: left-ventricular ejection fraction; LVFS:left-ventricular percent fractional shortening. ^*^, *P* < 0.01 *vs*. Sham+NS group, n=8; ^#^, *P* < 0.01 *vs*. I/R+LNA-scr group, n=8. Sham+NS: sham + saline injection group; I/R+NS: ischemia reperfusion injury + saline injection group; I/R+LNA-scr: ischemia reperfusion injury + microRNA-126a-5p inhibitor control injection group; I/R+ LNA-microRNA-126a-5p: ischemia reperfusion injury + microRNA-126a-5p inhibitor injection group.

Further, we tested the effect of microRNA-126a-5p ablation on the left ventricular cardiac function by echocardiography measurements. To verify this effect, transthoracic echocardiography and M-mode tracings were used to evaluate LVIDd, LVIDs, LVFS% and LVEF%. As expected, I/R injury significantly increased the LVIDd and LVIDs (Figure 6A and 6B), while reduced LVFS% and LVEF% (Figure 6C and 6D) relative to sham group. No significant difference was seen in the heart rate in any group (data not shown). Next, the mice pre-treated with miR-126a-5p inhibitor displayed significantly reduced LVIDd and LVIDs, relative to I/R-injured mice, as shown in Figure 6A and 6B. Conversely, the LVFS% and LVEF% increased after treatment with microRNA-126a-5p inhibitor (Figure 6C and 6D). However, the scramble/control inhibitor had no effect on I/R-induced cardiac injury.

### Hspb8 is a direct target gene of microRNA-126a-5p

**Figure 7:**
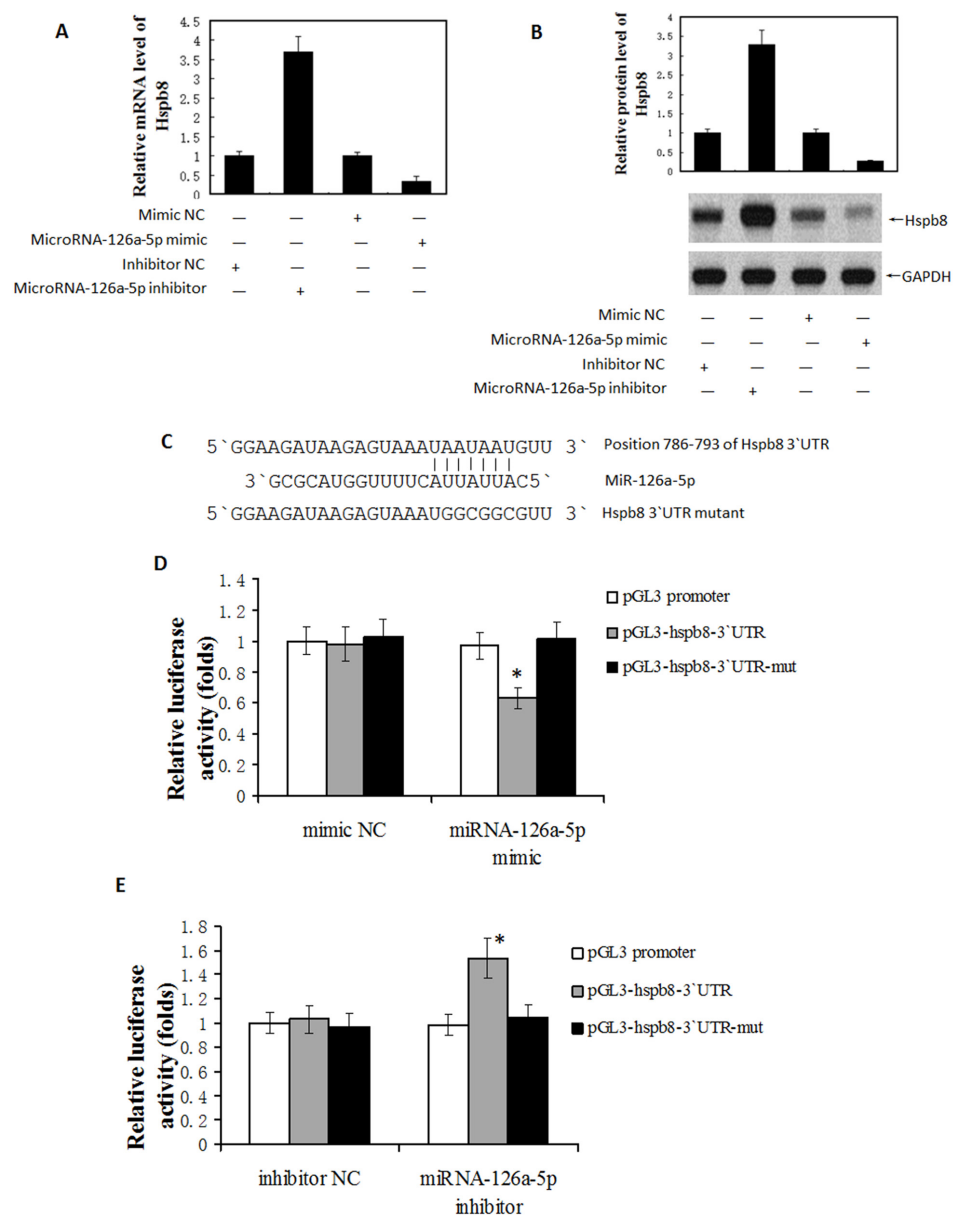
MicroRNA-126a-5p downregulated Hspb8 expression by binding to its 3′-untranslated region (3`UTR) **(A)** Hspb8 expression was significantly decreased by microRNA-126a-5p mimic while increased by microRNA-126a-5p inhibitor at mRNA level. (n = 3, ^*^P < 0.05). **(B)** Hspb8 protein expression was decreased by microRNA-126a-5p mimic while increased by microRNA-126a-5p inhibitor at protein level. **(C)** Schematic diagrams of the luciferase reporter construction. Wild type (pGL-hspb8-3`UTR) and mutant (pGL-hspb8-3`UTR-mut) 3` UTR of Hspb8 containing the binding site with microRNA-126a-5p were inserted into pGL promoter vector. **(D, E)** HEK293 cells were transfected with microRNA-126a-5p mimic (D)/inhibitor(E) combined with luciferase reporter (Wt). The effect of microRNA-126a-5p on the luciferase activity was measured by luciferase reporter assays. MicroRNA-126a-5p could suppress while its inhibitor promoted the luciferase activity. On the other hand, the microRNA-126a-5p binding sites were mutated and the mutated luciferase reporters were co-transfected with microRNA-126a-5p mimic/inhibitor. The mutations on binding sites abolished the previously suppressive or promoted effects. (n = 3 each group, ^*^P < 0.05).

It is well known that microRNAs exert their functions through suppressing target gene expression. Several bioinformatic websites, such as PicTar (http://pictar.mdc-berlin.de/), TargetScan (http://www.targetscan.org/) and miRBase (http://www.mirbase.org/) were used to predict the targets of microRNA-126a-5p. Among the candidates predicted by bioinformatics analysis, we found that Hspb8 is a potential target of microRNA-126a-5p because the seed sequences of microRNA-126a-5p align perfectly with the 3`-UTR of Hspb8 mRNA (Figure 7A). By transfecting H9C2 cells with miRNA mimics and inhibitors, we demonstrated microRNA-126a-5p mimic (M) significantly downregulated the mRNA and protein levels of Hspb8 and the expression of Hspb8 was significantly upregulated in microRNA-126a-5p inhibitor (I) group (Figure 7B and 7C).

Next, reporter constructs containing either wild-type (WT) Hspb8 3`-UTR or mutated Hspb8 3`-UTR (MT) at the predicted microRNA-126a-5p target sequences was transfected into 293T cells and then co-transfected with mimic-NC, microRNA-126a-5p mimic, microRNA-126a-5p inhibitor or inhibitor-NC. Luciferase reporter assays showed that microRNA-126a-5p mimic significantly decreased the luciferase activity of the WT Hspb8 3`-UTR by approximately 37.3% in 293T cells relative to the control (p < 0.05, Figure 7D), whereas microRNA-126a-5p inhibitor substantially increased luciferase activities of WT Hspb8 3`-UTR compared with inhibitor-NC (p < 0.05, Figure 7E). These results demonstrate that Hspb8 is the direct target of microRNA-126a-5p.

### Hspb8 siRNA inhibited the microRNA-126a-5p inhibitor-mediated protection against H_2_O_2_-induced injury

**Figure 8:**
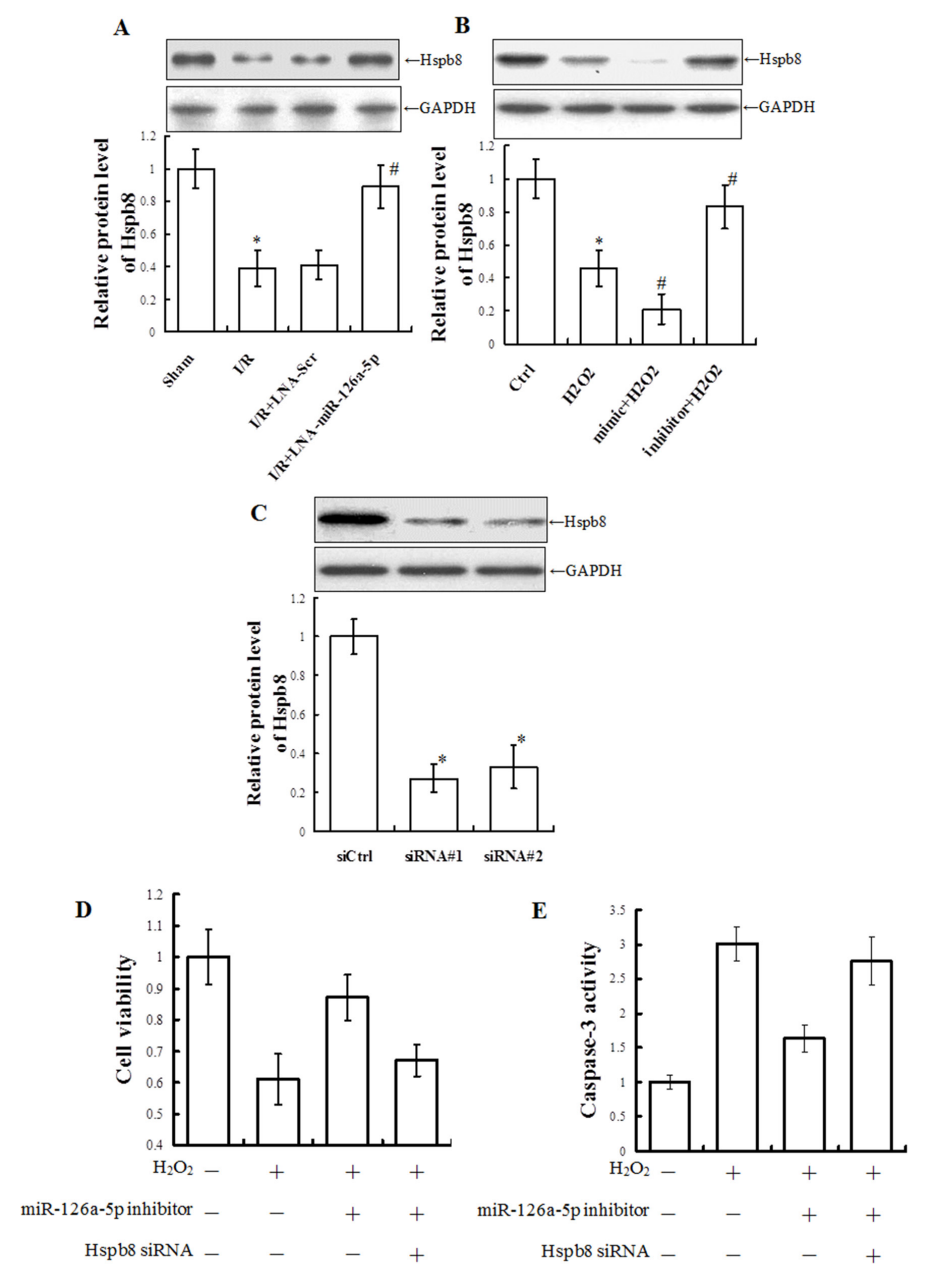
Hspb8 siRNA inhibited microRNA-126a-5p inhibitor-mediated protection against H_2_O_2_-induced injury in H9C2 cells **(A)** Western blot analysis of Hspb8 levels in total tissue extracts from infarct zone tissue treated with microRNA-126a-5p inhibitor (LNA-miR-126a-5p) or control inhibitor (LNA-Scr). ^*^, *P* < 0.05, *vs*. Sham group, n=6; ^#^, *P* < 0.05, *vs*. I/R+LNA-scr group, n=6. **(B)** Western blot analysis of Hspb8 levels in total cell extracts prepared from H9C2 cells treated with microRNA-126a-5p mimic or inhibitor. ^*^, *P* < 0.05, *vs*. Ctrl group, n=8; ^#^, *P* < 0.05, *vs*. H_2_O_2_ group, n=8. **(C)** Analysis of Hspb8 levels total cell extracts prepared from H9C2 cells transfected with control siRNA (siCtrl) or HSPB8-specific siRNAs. siRNA #1: #195633; siRNA #2: #195635. ^*^, *P* < 0.05, *vs*. siCtrl group, n=4. **(D, E)** Analysis ofcell viability (D) and caspase 3 activity (E) in H9C2 cells exposed to H_2_O_2_ for 24h after co-transfection with microRNA-126a-5p inhibitor and Hspb8 siRNA #1 for 24h. ^*^, *P* < 0.05, *vs*. H_2_O_2_ group, n=6; ^#^, *P* < 0.05, *vs*. inhibitor+H_2_O_2_ group, n=6.

Our finding that Hspb8 is a direct target gene of microRNA-126a-5p raised the possibility that Hspb8 levels may be the intermediate between microRNA-126a-5p and M-I/R injury. To test this possibility, we investigated the effect of microRNA-126a-5p on Hspb8 expression in the animal and cell model of I/R injury. Western blot analyses demonstrated that Hspb8 expression was down-regulated significantly after reperfusion 12h, and treatment with microRNA-126a-5p inhibitor (LNA-miR-126a-5p) inhibited the down-regulation of Hsbp8 *in vivo* (Figure 8A). Similarly, after exposure to H_2_O_2_ for 24h, Hspb8 expression was down-regulated significantly and further decreased in the cells transfected with microRNA-126a-5p mimic, but the change was reversed by microRNA-126a-5p inhibitor (Figure 8B).

To further confirm a role for Hspb8, Hspb8-specific siRNAs were used to inhibit Hspb8 expression. Western blot analysis showed H9C2 cells transfected with Hspb8-specific siRNAs achieved ∼73% (#1) and ∼67% (#2) depletion of Hspb8, respectively (Figure 8C). Then the protection role of microRNA126a-5p inhibitor was observed after Hspb8 ablation. Consistent with the role as a target molecule of microRNA126a-5p, depletion of Hspb8 relieved the protective role of microRNA126a-5p inhibitor. Cell viability was robustly decreased after H_2_O_2_ treatment. Treatment with microRNA-126a-5p inhibitor significantly rescued H_2_O_2_-induced decrease in cell viability in H9C2 cells (*p* < 0.05), however, treatment with Hspb8-specific siRNA (#1) relieved the increase in cell viability mediated by microRNA-126a-5p inhibitor (Figure 8D). Moreover, caspase 3 activity was significantly increased following H_2_O_2_ exposure, and microRNA-126a-5p inhibitor reversed the increase of caspase 3 activity. However, Hspb8-specific siRNA (#1) inhibited the changes of caspase 3 activity mediated by microRNA-126a-5p inhibitor (Figure 8E).

## DISCUSSION

Myocardial ischemia and reperfusion (I/R) injury is a phenomenon where blood supply to the heart is restricted momentarily and then restored back, but the damages to the tissues and organs may not recover completely. This is a complex pathophysiological event involving various mechanisms. The myocardial I/R injury induces the myocardial damage including cell death, cardiomyocyte hypertrophy and angiogenesis. There are many factors that can lead to cell death, such as pH abnormality [16], cellular calcium overload [17], oxidative stress [18], and release of inflammatory mediators [19]. The death of cardiomyocytes causes heart dysfunction and may eventually lead to heart failure. Thus, it is important to find some effective ways to prevent cell death from M-I/R injury. In our study, we observed that M-I/R-mediated injury to heart is mainly reflected in disorderly arrangement and dissolvement of myocardial fibers, infiltration by inflammatory cells and the increase in serum LDH, CK-MB levels.

MicroRNAs function as gene silencers and are emerging as important regulators for gene expression and biological processes. Recently, several studies have suggested that miRNAs are involved in cardiac events such as muscle contraction, heart growth and conductance of electric signal [20]. They play an important role in myocardial remodeling [21], arrhythmia [1], atherosclerosis [22] and myocardial regeneration [23]. It has been demonstrated that upregulation of miR-22 contributes to M-I/R injury by interfering with the mitochondrial function [24]. MicroRNA-128 inhibitor attenuates the apoptosis of cardiomyocytes during M-I/R injury through the activation of peroxisome proliferator-activated receptor gamma [25]. Moreover, miR-93 inhibits ischemia reperfusion-induced cardiomyocyte apoptosis by targeting PTEN [26]. MiR-195 may promote cardiomyocyte apoptosis by targeting Bcl-2 and inducing mitochondrial apoptotic pathway during ischemia reperfusion-induced myocardial injury [27]. Considering that multiple miRNAs play important roles in M-I/R injury, it is necessary to identify the differentially expressed microRNAs during myocardial I/R injury. In this study, a comparative miRNA profiling was performed in myocardium between sham group and I/R group. In response to I/R injury, remarkable change of miRNAs in myocardium was observed. MicroRNA-126a-5p was identified as one of the differentially expressed miRNAs compared to its expression in normal heart tissue.

MicroRNA-126a-5p is one of the miRNAs located on the intron of epidermal growth factor like domain 7 gene [13]. The *in vivo* and *in vitro* studies have indicated that microRNA-126a-5p plays crucial roles in maintaining the integrity of blood vessels, the formation of new blood vessels and repairing of the damages [28–34]. Recently, Tang *et al.* found down-regulation of microRNA-126a-5p leads to overexpression of VEGFA in lipopolysaccharide-induced acute lung injury [35]. But the function of microRNA-126a-5p in myocardial I/R injury is still unknown.

In this study, we observed the expression of microRNA-126a-5p in M-I/R injury mouse model and RT-QPCR results revealed that I/R injury increased the expression of microRNA-126a-5p. In parallel, we observed that H_2_O_2_ or H/R-induced injury in H9C2 cardiomyocytes also increased microRNA-126a-5p expression. To further study whether microRNA-126a-5p is involved in mediating H_2_O_2_-induced cardiac muscle cell injury or not, we modulated microRNA-126a-5p expression by using its mimic and inhibitor. Upregulation of microRNA-126a-5p expression via its mimic promoted the H_2_O_2_-induced apoptosis and the release of LDH. Conversely, H_2_O_2_-induced apoptosis and LDH release were inhibited by down-regulation of microRNA-126a-5p expression by microRNA-126a-5p inhibitor. These results clearly suggest that microRNA-126a-5p can inhibit cell activity and promote cell apoptosis.

To further validate the observation, we investigated the role of microRNA-126a-5p during M-I/R injury *in vivo*. Injection of microRNA-126a-5p inhibitor to mice through tail vein led to the reduction of its endogenous expression. This allowed us to study the role of microRNA-126a-5p during M-I/R injury. Down-regulation of microRNA-126a-5p expression by its inhibitor in mice with I/R injury could reduce I/R injury-mediated CK-MB expression and myocardial caspase-3 activity. Similarly, it also resulted in the improvement of hemodynamic indexes and cardiac function. Thus, lower expression of microRNA-126a-5p can attenuate the effects triggered by M-I/R injury.

MicroRNAs play a critical role in various biological activities through post-transcriptional suppression. By using online bioinformatics tools, Hspb8 was predicted to be a target gene of microRNA-126a-5p and we further confirmed this prediction by using biological assays. Hspb8, also named heat shock protein 22/H11 Kinase (Hsp22), is the stress-inducible small heat shock protein responsive to various conditions of myocardial stress, including ischemia [36]. Hspb8 is highly expressed in cardiac tissue [37]. Cardiac-specific overexpression of Hspb8 in a transgenic mouse provides protection against myocardial ischemia that is equally powerful to ischemic preconditioning through the induction of the inducible isoform of nitric oxide synthase (iNOS) [38].

In summary, our results demonstrated that microRNA-126a-5p expression was up-regulated during M-I/R injury. Over-expression of microRNA-126a-5p inhibited cell viability and promoted cell apoptosis, while the inhibition of microRNA-126a-5p protected the myocardium from the damages during M- I/R injury *in vitro* and *in vivo* through targeting Hspb8. These findings not only provide solid evidence for the mechanisms of myocardial I/R injury but also provide preclinical evidence that microRNA-126a-5p may be considered as a new therapeutic target to intervene the myocardial I/R injury.

## MATERIALS AND METHODS

### Materials

Adult male wild-type C57BL/6 mice weighing about 18-22 g were purchased from the Animal Center, Central South University (Changsha, China). The mice were housed in a temperature- and humidity-controlled animal room at 25°C with a 12:12h day/night cycle. H9C2 cardiomyocyte cell line was purchased from American type culture collection (ATCC) and grown in Dulbecco’s Modified Eagle (DMEM) medium supplemented with 10% fetal bovine serum at 37°C with 5% CO_2_. Syn-mmu-microRNA-126a-5p miScript miRNA Mimic (Code: MSY00013), anti-mmu-126-5p miScript miRNA inhibitor (CodeMIN000137), AllStar negative control siRNA (Code: 1027280), miScript inhibitor negative control (Code: 1027271) and Hiperfect transfection reagent were purchased from Qiagen Corporation. MiRCURY LNA (Locked Nucleic Acid) microRNA-126a-5p mouse inhibitor was procured from Exiqon (Vedbaek, Denmark). The sequences of miRCURY LNA microRNA-126a-5p inhibitor are 5`-CGCGTACCAAAAGTAATAATG-3`and the sequences of miRCURY LNA control microRNA inhibitor are 5`-GTGTAACACGTCTATACGCCCA-3`. DEPC, H_2_O_2_ and sodium dithionite (Na_2_S_2_O_4_) were purchased from Sigma Corporation (St. Louis, MO). Caspase-3 and LDH kits were purchased from Beyotime Corporation (Shanghai, China). Negative control-siRNA (Silencer^®^ Negative Control #1 siRNA), Hspb8-siRNAs (Silencer^®^ siRNA, #195633 and #195635) were obtained from Thermo Fisher Scientific, Corp. (Frederick, MD, USA).

### Methods

#### Development of *in vivo* mouse model for M-I/R injury

All adult male C57BL/6 mice with similar body weight were anesthetized by intraperitoneal (i.p.) injection of ketamine (100 mg/kg) and xylazin (Rompun 10 mg/kg) before endotracheal intubation. Then mouse’s thoracic cavity was opened by left thoracotomy, and 8-0 prolene suture was passed under the left anterior descending (LAD) coronary artery located at the inferior edge of the left atrium and tied to produce an occlusion. After LAD coronary artery ligation, the persistent elevation of the ST segment was located. This led to the successful confirmation of the ischemia model. Post 30 mins of ischemia induction, the ligature was released and mice were reperfused for different time points. At last, the chest of mouse was closed with 6-0 prolene sutures and endotracheal tube was removed to resume spontaneous respiration. In addition, the sham group mice were operated similarly, except the ligation of the left anterior descending coronary artery. At the time of sacrifice, anesthesia was induced by isoflurane (3% induction, 1.5% maintenance). The myocardial tissues from the infarct zone and blood were harvested for morphology, quantitative and qualitative analysis.

### Hematoxylin-eosin staining

The myocardial tissues from the infarct zone were quickly collected and fixed with 4% neutral-buffered paraformaldehyde for 24 hrs, according to the standard procedure. The 5mm thick sections were embedded in paraffin. Next, H&E staining was used to examine the change in myocardial tissue morphology. These stained sections were examined under optical microscope to assess the degree of heart damage and subsequently photographed.

### Assessment of creatine kinase and LDH in the serum

The assessment of creatine kinase and LDH serum levels was performed as described previously [40, 41].

### Sample collection and total RNA extraction

The myocardial tissues from the infarct zone were immediately frozen in the liquid nitrogen. Then the samples were homogenized in RNase-free mortars with liquid nitrogen and TRIzol reagent (Invitrogen, USA), and total RNA was extracted.

### MicroRNA microarray

Sample labeling, microarray hybridization, and wash were performed based on the manufacturer’s standard protocols (Agilent Technologies Inc., Santa Clara, California, USA). After dephosphorylation and denaturation, total RNA was labeled with cyanine 3-pCp and then hybridized to Agilent miRNA Microarray V2.0. Following hybridization for 20 hrs, the slides were washed using the Gene Expression Wash Buffer Kit (Agilent) and scanned using an Agilent Scanner. The images were processed and analyzed with Agilent Feature Extraction Software. The raw data were normalized using quantile normalization and then analyzed by GeneSpring GX software (zcomSilicon Genetics, Redwood City, CA, USA). Statistical analysis was conducted by using ANOVA to compare the differentially expressed miRNAs. The threshold set for significantly up- and down-regulated genes was fold change >2.0 and P value < 0.05.

### Quantitative real-time PCR for microRNA-126a-5p expression

Total RNA was isolated from the heart tissues and H9C2 cells using the miRNeasy mini kit (Qiagen). The miScript reverse transcription kit (Qiagen) and miScript SYBR Green PCR kit (Qiagen) were used to measure the expression levels of microRNA-126a-5p with its specific primers and the miScript Universal Primer (Qiagen), using a Fast Real-Time PCR system, model 7500 (Applied Biosystems, Foster City, CA, USA). All reactions were incubated at 95°C for 30 s, followed by 40 cycles of 95°C for 5 s and 60°C for 34 s Expression level of the U6B small nuclear RNA (RNU6B) was used as an endogenous control to normalize the sample data. Relative expression levels were calculated with the 2^–ΔΔCt^ method. Every experiment was repeated at least three times.

### Treatment of H9C2 cells with H_2_O_2_

H9C2 cells (1×10^4^-1×10^5^) were seeded into each well of 96-well plates. They were treated with various concentrations (0 mg/L, 0.25 mg/L, 0.5 mg/L, 0.75 mg/L, 1 mg/L) of H_2_O_2_ for 24 hrs or treated with 0.5 mg/L of H_2_O_2_ for different time points (0 hr, 6 hrs, 12 hrs and 24 hrs).

### Preparation of cardiomyocyte hypoxia/reoxygenation model

Cardiomyocytes hypoxia/reoxygenation model was prepared by Na_2_S_2_O_4_ as described previously [39] with some modifications. The H9c2 cells were exposed to Na_2_S_2_O_4_ at a concentration of 4mM in the low glucose DMEM medium for 4h at 37°C and 5% CO_2_, causing hypoxia-induced damage. Then, the medium was replaced with normal medium (high glucose) for 12 h at 37°C and 5% CO_2_. The viability of H9C2 cells treated with Na_2_S_2_O_4_ was assessed by MTT assay.

### Cell transfection

H9C2 cells were transfected with microRNA-126a-5p mimic or inhibitor according to the manufacturer’s protocols. Firstly, 1.0×10^5^ cells were seeded in each well of 24-well plate and cultured under normal growth conditions (typically 37°C and 5% CO_2_). Next, 0.15 μl of miRNA mimic or 1.5 μl of miRNA inhibitor was diluted in 100 μl culture medium without serum, followed by the addition of 3 μl of HiPerFect transfection reagent to the diluted miRNA mimic/inhibitor mixture by vortexing. The mixture was incubated for 5-10 mins at room temperature and added drop-wise onto the culture medium. Finally, cells were incubated with this transfection mixture under normal growth conditions for 24 hrs and microRNA-126a-5p expression was detected by RT-QPCR. To knockdown Hspb8 in H9C2 cells, the cells were transfected with Hspb8 siRNAs (#195633 and #195635) or the negative control siRNA using HiPerFect transfection reagent in accordance with the manufacturer’s instructions. After transfection for 48h, Hspb8 protein expression was analyzed by Western blot.

### Cell viability, caspase 3 activity assay and western blot

The caspase activity assay, cell viability assay, and western blot were performed as described previously [40, 41]. Rabbit monoclonal anti-hspb8 antibody (ab151552, Abcam) and Rabbit monoclonal anti-GAPDH antibody (2118L, cell signaling) were used.

### Knockdown of microRNA-126a-5p in mice

Healthy male C57BL/6 mice weighing 18-22 g were randomly divided into four groups: (1) the sham+NS group (n=8): these mice underwent sham operation without coronary artery ligation and received normal saline injection via tail vein; (2) the I/R +NS group (n=8): the mice received ischemia induction for 30 min followed by 12 hrs reperfusion, and were then injected with normal saline via tail vein; (3) the I/R + microRNA-126a-5p inhibitor control group (n=8): these mice underwent ischemia induction for 30 min followed by 12 hrs reperfusion, and were injected with microRNA-126a-5p control inhibitor via tail vein; (4) the I/R + microRNA-126a-5p inhibitor group (n=8): the mice underwent ischemia induction for 30 min followed by 12 hrs reperfusion, and were then injected with microRNA-126a-5p inhibitor via tail vein. After 12 hrs of these control or inhibitor injection, we performed the echocardiography and cardiac haemodynamic measurements to assess the heart function. After measurements, the mice were sacrificed to collect the heart tissues and serum.

### Echocardiography analysis

Mice were kept on a heating pad in a left lateral decubitus or supine position under isoflurane (2%) anesthesia and two-dimensional images were recorded. LV parameters including interventricular septum thickness, LV posterior wall thickness, LV internal diastolic diameter (LVIDd) and LV internal systolic diameter (LVIDs) were obtained from M-mode interrogation in a long-axis view. The LV percentage fractional shortening (LV%FS) and LV ejection fraction (LVEF) were calculated as follows: LV%FS = (LVIDd − LVIDs)/LVIDd × 100; and LVEF = [(LVIDd)3 − (LVIDs)3]/(LVIDd)3 × 100. All echocardio-graphic measurements were averaged from at least three separate cardiac cycles.

### Hemodynamic measurements

Hemodynamic measurements were performed in mice after I/R. Mice were kept under isoflurane (2%) anesthesia. A Millar catheter (SPR-1000) was inserted through the right carotid artery into the LV. The LV systolic pressure (LVSP), LV end-diastolic pressure (LVEDP), maximum rate of LVSP increase (+dp/dt max) and decrease (−dp/dt max) were recorded and analyzed using a PowerLab data acquisition system (model ML866; ADInstruments, Colorado Springs, CO).

### MicroRNA target prediction

To predict the target genes of miR-126a-5p, bioinformatics algorithms TargetScan (http://www.targetscan.org) miRBase (http://www.mirbase.org/) and TargetScan (http://www.Targetscan.org/) were used.

### Hspb8 3′-UTR cloning and luciferase assay

Luciferase reporter assay was performed as described previously [40]. A fragment of the Hspb8 3′-UTR containing the predicted binding site or their mutant fragment sequence on each side with suitable enzyme cleavage sites were synthesized and cloned into the downstream of luciferase reporter gene (Hspb8 Wt-Luc or Hspb8 Mu-Luc). Each vector, along with Relina vector and microRNA-126a-5p mimics or negative control or microRNA-126a-5p inhibitor, anti-miR-126a-5p or Anti-NC, were transfected into 293T cells using Lipofectamine 2000 reagent (Invitrogen, USA) following the instructions. Cells were harvested 48 h after transfection and luciferase activity was detected using the Dual-Luciferase Reporter Assay System (Promega, USA).

### Statistical analysis

The data were represented as mean±SEM and all statistical analyses were performed using with GraphPad Prism 5 (GraphPad Software, Inc., San Diego, CA, USA). The differences between two groups were analyzed using unpaired Student’s t-test, while the differences among three or more groups were analyzed using one-way ANOVA (analysis of variance) followed by Student-Newman-Keuls.
